# The effect of ocular biometric factors on the accuracy of various IOL power calculation formulas

**DOI:** 10.1186/s12886-017-0454-y

**Published:** 2017-05-02

**Authors:** Jinho Jeong, Han Song, Jimmy K. Lee, Roy S. Chuck, Ji-Won Kwon

**Affiliations:** 1 Department of Ophthalmology, Jeju National University College of Medicine, Jeju, Korea; 2 Department of Ophthalmology, Myongji Hospital Seonam University College of Medicine, 697-24, Hwajung-Dong, Deokyang-Gu, Goyang-Si, Gyeonggi-Do 412-270 Korea; 3 Department of Ophthalmology and Visual Sciences, Montefiore Medical Center, Albert Einstein College of Medicine, New York, USA

**Keywords:** Anterior chamber depth, IOL formula, Ocular biometry

## Abstract

**Background:**

To evaluate how differences in ocular biometry affects the Hoffer Q, Holladay 1, SRK/T, and Haigis intraocular lens power calculation formulae predictions.

**Methods:**

This study was performed on 91 eyes of 91 patients who underwent uneventful cataract surgery. Ocular biometry values were measured using the IOL Master 500, and intraocular lens (IOL) power was calculated using the Haigis, Hoffer Q, Holladay 1, and SRK/T formulas. We calculated the expected difference (ED) of each 3rd generation formula from the Haigis formula by subtracting the predicted refraction of the Haigis formula from the predicted refraction of each 3rd generation formula. Post-operative anterior chamber depth (ACD) was measured at 1 month after surgery using the IOL master. We calculated errors of each formula by subtracting predicted from manifest refraction at post-operative 1 month. Correlation analysis was performed between ocular biometry values, formula expectation values, formula errors and absolute formula errors.

**Results:**

Multiple regression analysis revealed that preoperative ACD was the only significant factor for ED prediction in all of the 3rd generation formulas. For mean errors, axial length and post-operative 1-month change of ACD (delta ACD) correlated significantly with the errors in all 3rd generation formulas, but not with errors of the Haigis formula. Median absolute error (MedAE) of the formulas were 0.40 D for the Hoffer Q formula, 0.37 D for the Holladay formula, 0.34 D for the SRK/T formula, and 0.41 D for the Haigis formula. The MAE of the formulas were 0.50 ± 0.47 D for the Hoffer Q formula, 0.50 ± 0.50 D for the Holladay formula, 0.47 ± 0.51 D for the SRK/T formula, and 0.50 ± 0.47 D for the Haigis formula.

**Conclusion:**

Regarding ED between the third generation and Haigis formulas, preoperative ACD demonstrated the greatest influence. Calculating mean absolute errors of the formulas, all IOL formulas showed excellent and comparable accuracy. Post-operative change (delta) of ACD correlated significantly with errors of third generation formulas according to simulated ACD.

## Background

Cataract surgery can certainly result in postsurgical refractive outcomes different from anticipated refractive errors [1]. Among the wide range of intraocular lens power formulas available, the Haigis formula has garnered attention by incorporating the preoperative measurement of the anterior chamber depth using laser interferometry [2]. However, immersion ultrasound biometry is also regularly used, particularly in settings where laser interferometry is unavailable, in cases of mature or subcapsular cataracts, or if the patient is unable to remain seated [3]. Therefore, third generation theoretic formulas, such as Hoffer Q and SRK/T formulas, still have much clinical importance [4–7].

The third-generation formulas estimate postoperative effective lens position (ELP) by using various preoperative biometric variables such as central corneal power and axial length [3]. Different consideration of variables in each formula can lead to disparities in ELP estimation and predicted postoperative refractions [8]. Most studies on the accuracy of IOL formulas compare the degree of error between formula-predicted refraction and measured post-operative refraction, seldom searching for characteristics of direct correlations of predicted refraction difference between formulas with various combination of pre-operative biometric values [9, 10]. It is not unusual for surgeons to cross-reference different IOL formulas; if different formulas predict values that are widely divergent, confusion regarding the choice of the IOL may arise. In this study, we investigated situations whereby the predicted refraction of the Haigis, Hoffer Q, Holladay 1 and SRK/T formulas varied widely and attempted to compare the accuracies of these formulas.

## Methods

A retrospective review was conducted of uneventful cataract surgeries that were performed on 91 eyes by a single surgeon (J.J.) using the same technique. This study was performed according to the Declaration of Helsinki on Biomedical Research Involving Human Subjects. The Institutional Review Board of the Jeju university hospital approved the clinical study. Patients provided written informed consent to participate after being given a detailed explanation of the study. Preoperative manifest refraction was recorded. Biometric measurements of keratometry, anterior chamber depth (ACD), and axial length of each eye were taken using the IOL master 500 (Carl Zeiss Jena, Germany). ACD measurement was taken using the lens-referenced method, which is the distance from the epithelium to the anterior pole of the crystalline lens or IOL [11]. All measurements were taken at least three times by an experienced technician and the data was averaged after three reproducible readings were obtained. Patients who had previously undergone corneal refractive surgery or who had irregular surface diseases, including pterygium or scarring, severe dry eye syndrome with corneal erosions, or who had intraoperative complications, such as posterior capsular tear, were excluded from study.

From the axial length and corneal curvature data, IOL power was calculated using the Haigis, Hoffer-Q, Holladay 1 and SRK/T formulas, using the IOL master software. We calculated the expected difference (ED) of each 3rd generation formula from the Haigis formula by subtracting the expected refraction of the Haigis formula from that of the 3rd generation formula:

$$ \mathrm{ED}=\mathrm{Expected}\ \mathrm{fraction}\ \mathrm{of}\ \mathrm{each}\;{3}^{\mathrm{rd}}\;\mathrm{generation}-\mathrm{expected}\ \mathrm{refraction}\ \mathrm{of}\ \mathrm{the}\ \mathrm{Haigis}\ \mathrm{formula} $$

We performed regression analysis of the ED with the pre-operative biometric factors to evaluate the cause of formula expectation differences.

Based on preoperative keratometry values, the steep meridian of the cornea was determined. Phacoemulsification was performed through a 2.2 mm, clear corneal incision along the steep axis using a phacoemulsification system (Infinity system) (Alcon, Fort Worth, TX, USA). Following phacoemulsification, a hydrophobic, acrylic 1-piece IOL (Tecnis) (AMO, Santa Ana, CA, USA), with an A-constant of 118.7 was implanted into the capsular bag. Post-operative ACD was measured 1 month after surgery. Determination of refractive outcome was made 1 month after surgery by determining the error associated with each formula’s predicted IOL power. The error associated with each formula was calculated by subtracting the spherical equivalent of the manifest refraction from the expected refraction determined by each formula. The errors between the formulas were correlated with pre-operative biometric factors and post-operative 1 month change of ACD. The mean error (ME) and the mean absolute error (MAE) of each formula were calculated and correlated with biometric factors. MAE of each formula was calculated and compared using the Friedman non-parametric test. Statistical analyses were conducted using SPSS statistical software (version 21.0, SPSS Inc., Chicago, IL); *p*-value <0.05 was considered statistically significant.

## Results

This study included unilateral 91 eyes of 91 patients (M:F = 51:40) with a mean age of 70.7 years. The axial length of eyes ranged from 21.61 to 27.91 mm (mean = 23.73 mm) with a normal distribution. Other biometric parameters including preoperative refractive error, corneal curvature, and anterior chamber depth are summarized in Table 1. The mean value of preoperative ACD was 3.13 mm, and the mean ACD measured at post-operative 1 month was 4.49 mm.

**Table 1:** Characteristics of preoperative biometric data and the power of IOL distribution

	Minimum	Maximum	Mean
Age (year)	47	81	70.71 ± 8.99
Axial length (mm)	21.61	27.91	23.73 ± 1.55
Cylinder of refractive error (diopter)	0	3.00	1.17 ± 0.73
Corneal curvature (diopter)	40.75	47.25	43.75 ± 1.60
Pre-op Anterior chamber depth (mm)	2.15	4.05	3.13 ± 0.49
Post-op Anterior chamber depth (mm)	2.75	5.50	4.49 ± 0.50

**Fig. 1:**
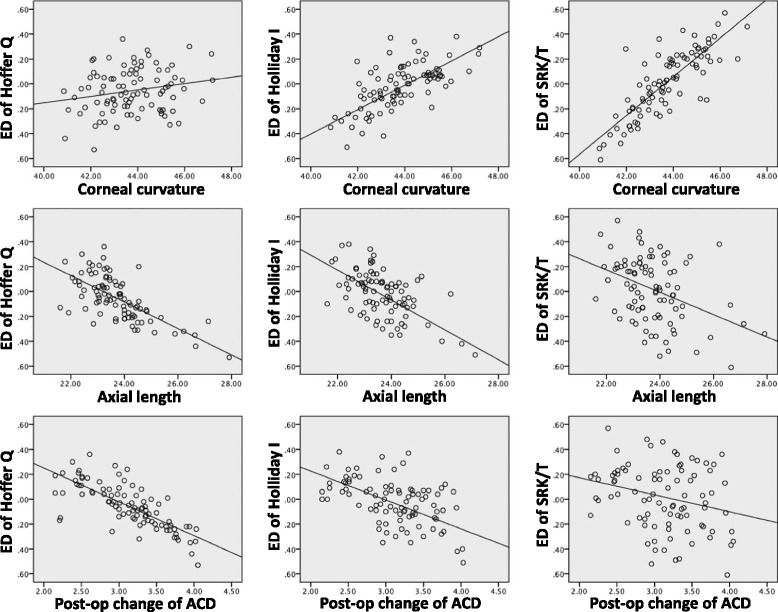
The scatterplot of preoperative biometric values with expected difference (ED) of the 3rd generation formulas from the Haigis formula

The percentage of cases showing ED within 0.4 D was 97.8% for the Hoffer Q formula, 95.6% with the Holladay formula, and 93.41% the SRK/T formula. Bivariate correlation analyses of several preoperative parameters for the ED of the formulas were performed (Fig. 1). Cornea curvature was significantly correlated with the ED of SRK/T (*r* = 0.827) and Holladay (*r* = 0.665) formulas, but not with Hoffer Q (*r* = 0.206, *p* = 0.05). Axial length was significantly correlated with the ED of Hoffer Q (*r* = −0.681), Holladay (*r* = −0.653) and SRK/T (*r* = −0.382) formulas. Preoperative ACD was also significantly correlated with the ED of Hoffer Q (*r* = −0.756), Holladay (*r* = −0.548) and SRK/T (*r* = −0.252) formulas (Table 2). Multiple regression analysis revealed that pre-operative ACD was the only significant factor for the ED with all 3rd generation formulas. Corneal curvature was not a significant factor for the ED of the Hoffer Q formula (*p* = 0.809), and the axial length was not a significant factor for the ED of the Holladay formula (*p* = 0.072) (Table 3).

**Table 2:** The correlation analysis of biometric values for the expected difference (ED) of each 3rd generation formulas from the Haigis formula

Pearson Correlation	ED of Hoffer Q	ED of Holladay	ED of SRK/T
Corneal curvature (D)	0.206 (*p* = 0.050)	0.665 (*p* = 0.000)	0.827 (*p* = 0.000)
Axial length (mm)	−0.681 (*p* = 0.000)	−0.653 (*p* = 0.000)	−0.382 (*p* = 0.000)
Anterior chamber depth (mm)	−0.756 (*p* = 0.000)	−0.548 (*p* = 0.000)	−0.252 (*p* = 0.016)
Delta ACD (mm)^a^	0.424 (*p* = 0.000)	0.201 (*p* = 0.056)	0.117 (*p* = 0.271)

^a^Delta ACD means post-operative 1 month change of anterior chamber depth (ACD)

**Table 3:** The results of multiple regression analyses of preoperative biometric values for the expected difference (ED) of each 3rd generation formulas from the Haigis formula

	ED of Hoffer Q	ED of Holladay	ED of SRK/T
Adjusted R^2^	0.659	0.715	0.747
Constant sig.^a^	0.000	0.000	0.000
Corneal curvature (D)	0.809	0.000	0.000
Axial length (mm)	0.000	0.072	0.005
Anterior chamber depth (mm)	0.000	0.000	0.000

^a^ *Constant sig.* = constant significance, *p* value of the multiple regression analysis

**Fig. 2:**
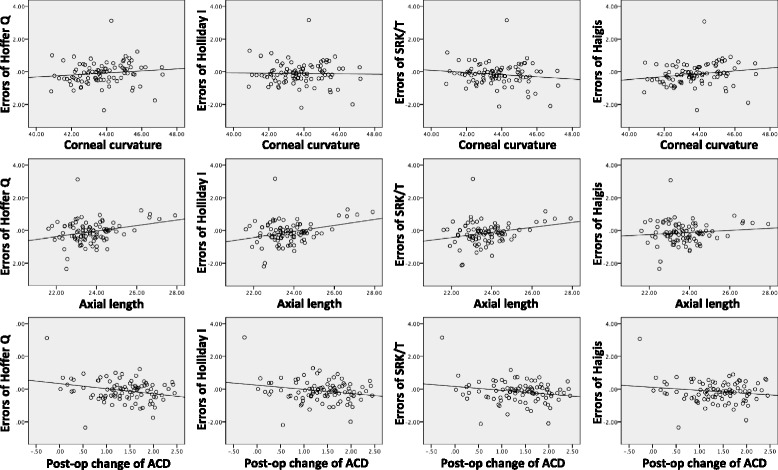
The scatterplot of preoperative biometric values with post-op mean errors of the formulas

By bivariate correlation analysis, the correlation of pre-operative and post-operative biometric values with the mean errors of the formulas were demonstrated (Fig. 2). Mean corneal curvature did not show significant correlation with the errors of any formulas. Mean axial length showed significant correlation with the errors of the Hoffer Q (*p* = 0.008), Holladay (*p* = 0.004), and SRK/T formulas (*p* = 0.013), but not with the Haigis formula (*p* = 0.303). Post-operative 1 month change of ACD (delta ACD) showed significant correlations with the errors of the Hoffer Q (*p* = 0.011), Holladay (*p* = 0.037), and SRK/T (*p* = 0.047) formulas, but not with Haigis formula (*p* = 0.120) (Table 4).

**Table 4:** The correlation analysis of preoperative biometric values for the difference of each formula expected value from the post-operative refraction

	Hoffer Q error	Holladay error	SRK/T error	Haigis error
Corneal curvature (D)	0.127 (*p* = 0.230)	−0.019 (*p* = 0.859)	−0.141 (*p* = 0.183)	0.187 (*p* = 0.077)
Axial length (mm)	0.277 (*p* = 0.008)^a^	0.301 (*p* = 0.004)^a^	0.259 (*p* = 0.013)^a^	0.109 (*p* = 0.303)
Pre-op ACD (mm)	0.265 (*p* = 0.011)^a^	0.238 (*p* = 0.023)^a^	0.176 (*p* = 0.096)	0.077 (*p* = 0.469)
Post-op change of ACD (mm)	0.266 (*p* = 0.011)^a^	0.219 (=0.037)^a^	0.209 (*p* = 0.047)^a^	0.164 (*p* = 0.120)

^a^Statistically significant

For the mean absolute errors (MAE) of the formulas, none of the preoperative biometric measurements showed significant correlations. Post-operative 1-month change of ACD (delta ACD) showed slightly stronger correlation with the MAE of all 3rd generation formulas, but none were statistically significant (Table 5).

**Table 5:** The correlation analysis of preoperative biometric values for mean absolute errors (MAE) of the formulas

	Hoffer Q MAE	Holladay MAE	SRK/T MAE	Haigis MAE
Corneal curvature (D)	0.019 (*p* = 0.860)	0.067 (*p* = 0.529)	0.155 (*p* = 0.142)	0.043 (*p* = 0.686)
Axial length (mm)	−0.016 (*p* = 0.880)	0.003 (*p* = 0.976)	−0.075 (*p* = 0.477)	−0.121 (*p* = 0.254)
Pre-op ACD (mm)	0.032 (*p* = 0.766)	0.061 (*p* = 0.565)	0.054 (*p* = 0.612)	0.002 (*p* = 0.983)
Post-op change of ACD (mm)	0.198 (*p* = 0.059)	0.191 (=0.069)	0.206 (*p* = 0.050)	0.180 (*p* = 0.088)

The MAE of the formulas were +0.50 ± 0.47 D for the Hoffer Q formula, +0.50 ± 0.50 D for the Holladay formula, +0.47 ± 0.51 D for the SRK/T formula, and +0.50 ± 47 D for the Haigis formula. The MAEs were compared between the formulas using the Friedman non-parametric test, and no statistically significant difference was observed (sig. = 0.747). MedAE of the formulas were 0.40 D for the Hoffer Q formula, 0.37 D for the Holladay formula, 0.34 D for the SRK/T formula, and 0.41 D for the Haigis formula.

All formulas produced similar percentage of MAEs within 1.0 D (98.2% (Hoffer Q formula), 98.0% (Holladay formula), 98.3% (SRK/T formula) and 98.4% (Haigis formula).

## Discussion

Various studies have reported that the Hoffer Q formula shows superior accuracy when evaluating eyes with short axial lengths, and that the SRK/T and Haigis formulas perform better for eyes with longer axial lengths [3, 12]. The consensus is that for average axial lengths (22–25 mm), all third-generation formulas demonstrate comparable accuracy for predicting refractive outcome.

We followed the protocols recently published in an Editorial in the American Journal of Ophthalmology, comparing IOL power calculation formulas typically evaluated the accuracy of the formula of interest by comparing the MAE or MedAE of each formula [13–15]. We did not perform an analysis stratifying according to axial length due to the limited number of study participants. There are differing opinions about the period necessary for post-operative refractive stability. According to some, post-operative refractive stability occurs by 6 weeks, while others report stability as early as 2 weeks [16]. We consider 1 month an acceptable period for post-operative refractive stability.

In our study, we analyzed the role of preoperative biometric parameters with the expectation disparities (ED) between the formulas. Corneal curvature, axial length, and ACD showed disparate contributions to the expected formula differences. For the ED of the Hoffer Q formula, axial length and ACD showed higher correlations. Pre-op ACD correlated more with the ED of Hoffer Q, which may be attributed to the Hoffer Q formula using a unique personalized ACD as a key variable when calculating IOL power. For the ED of the SRK/T formula, corneal curvature showed a stronger correlation (Table 2). We define ED to be the difference between the Haigis formula expectated value and each 3rd generation formula. A positive ED correlates with more hyperopic predicted refraction from each 3rd generation formula compared with the Haigis formula. In other words, positive EDs mean that target IOL power calculated by Haigis is lower than that calculated by 3rd generation formulas. ED was positively correlated with corneal curvature, and negatively correlated with axial length and pre-op ACD. Therefore, we deduce that there is a tendency that the post-operative refraction calculated by Haigis formula for a given IOL power results in myopic shift compared to 3rd generation formulas. Additionally, the ELP in smaller eyes (steep cornea, short AL, shallow ACD) is more anterior by the Haigis formula than in other 3rd generation formulas. Therefore, the three 3rd generation formulas may overestimate the ELP in short and steep eyes with shallow ACDs. Using multiple regression analysis, we found that preoperative ACD was the only significant factor in the ED difference between all 3rd generation formulas and the Haigis formula (Table 3). Significant differences in mechanism between the 3rd generation (SRK/T, Holladay I and Hoffer Q) and 4th generation (Haigis, Olsen and Holladay II) formulae may be considered with these results. Third generation formulas still use A constant based method to estimate ELP such as personalized ACD or surgeon factor, whereas Haigis formula is free from A constant.

For the post-op errors of IOL formulas, axial length and post-operative increase in ACD (delta ACD) were the most significant factors for all 3rd generation formulas, but not for the Haigis formula (Table 4). Haigis formula appeared to be less influenced by preoperative biometry changes and evidences a consistent level of formula error. Mean observed increase in post-operative 1 month ACD (delta ACD) was 1.36 mm (43.4%) using the single-piece Tecnis IOL with 5° posterior angulation. Similar to our findings, Kucumen et al. reported that using the single-piece Acysof IOL (Alcon, Fort Worth, TX, USA) with 6° posterior angulation, the post-operative increase in lens-referenced ACD was 1.37 mm (53.9%) at 1 month as measured by anterior segment OCT [11]. It also should be noted that delta ACD can vary between different IOLs depending on differences in vault angles and optic dimensions. However, because the effects of IOL angulation and dimensions are calculated and incorporated in unique A-constants from IOL manufacturers, IOLs with similar posterior angulations and A-constants demonstrate similar amounts of average delta ACD. Therefore, biometric factors such as zonular weakness and cataract maturity may have caused most of the delta ACD variance in such cases.

MAE of the formulas were similar among all formulas, and the percentage of MAE within 0.50 was also similar among all formulas. All the formulas were statistically comparable and excellent in accuracy.

Recently, Ladas et al. reported a novel method of combining multiple modern intraocular lens formulas to generate a super formula and maximize accuracy [17, 18]. Super surface was generated by connecting the most accurate part of each modern formula in a particular range of axial length and corneal curvature combination. The super surface was composed of the Hoffer Q below 22 mm, Holladay 1 between 22 and 25 mm, Holladay 1 with Koch adjustment over 25 mm, and Haigis in extreme long axial length and high corneal curvature conditions. However, it lacks consideration of ACD, and this current study may supplement understandings for the improvement of IOL accuracy by analyzing the influence of pre-operative and delta ACD on formulae outcomes.

The selection of IOL formula is most important in cases with very flat or steep corneas, very short or long axial lengths, and very shallow or deep ACDs. With multiple regression analysis, we determined that ACD variation was the only factor that affected the ED for all 3rd generation formulas. Secondly, for post-operative formula errors, we found that corneal curvature did not show any significant correlations, and that both axial length and delta ACD had significant correlations with mean errors for all 3rd generation formulas. In contrast, the errors associated with the Haigis formula remained uninfluenced by the aforementioned biometric factors. Therefore, in cases with a large expected ACD shift, the Haigis formula appears to be a better option.

The Haigis formula is the only one of the formulas which considers all three: corneal curvature, axial length, and pre-op ACD in its calculation of effective lens position. So, it is no surprise that the Haigis calculation is unaffected by variations in pre-op ACD, and that it is more sensitive to post-op ACD changes in cases where there is mature cataract, zonular weakness, or preoperative angle closure glaucoma, which may influence delta ACD.

The limitations of this study were that we had a relatively small number of cases, and lack of extreme corneal curvatures and axial lengths. We also did not incorporate other promising 4th generation formulas like the Holladay II or Barrett formulas because of availability. An important limitation of our study is that the postoperative refractive status was measured with manifest refraction in 0.25 diopter steps. This limitation may represent a significant source of error when considering the MAEs of approximately 0.5 diopter of this study.

## Conclusion

In summary, we found that accuracy was similar for the Haigis, Holladay, Hoffer Q, and SRK/T formulas. We achieved better understanding of how each variable in the formulas relatively weighed in each formula. Preoperative ACD was the key factor for the difference of all the 3rd generation formulas compared to Haigis formula. Axial length and the post-operative change (delta) of ACD showed significant correlations with the errors of the third-generation formulas.

## Abbreviations

ACD: Post-operative anterior chamber depth
CI: Confidence interval
Constant sig: Constant significance
MAE: The mean absolute error
MEDAE: Median absolute error
SE: Spherical equivalent
